# Exploratory Evaluation of *Chenopodium Chilense Schrad* for Gastrointestinal Parasite Control in Sheep

**DOI:** 10.3390/vetsci13060539

**Published:** 2026-05-29

**Authors:** David Cancino-Baier, Ximena Badilla A., Alex Muñoz, Camila Godoy S., Monserrat Aviles C., Matías Oñate, Rommy Diaz, John Quiñones, Nestor Sepulveda

**Affiliations:** 1 Facultad de Ciencias Agropecuarias y Medioambiente, Universidad de La Frontera, Temuco 4811230, Chile; 2 Carrera de Medicina Veterinaria, Universidad Mayor, Temuco 7500994, Chile; ximena.badilla@umayor.cl (X.B.A.);; 3 Programa de Doctorado en Ciencias Agroalimentarias y Medioambiente, Universidad de La Frontera, Temuco 4811230, Chile

**Keywords:** phytotherapy, small ruminants, fecal egg count, McMaster technique, botanical bioactive compounds, natural anthelmintics, helminth burden, ethnoveterinary medicine

## Abstract

Gastrointestinal parasitism remains a major constraint in sheep production, and increasing anthelmintic resistance has stimulated interest in plant-based alternatives. This exploratory study evaluated the antiparasitic effect and preliminary biochemical safety of *Chenopodium chilense Schrad*., a plant species, in sheep from southern Chile. Thirty adult female Criollo Araucana sheep were allocated to five groups (n = 6): two groups received commercial anthelmintics, and three groups received oral *C. chilense* at 0.5, 1.0, or 2.0 g of “Paico powder” per kg of body weight. Fecal egg counts were monitored over a three-week follow-up period using the McMaster technique. Serum AST, GGT, ALP, total protein, albumin, total cholesterol, and triglycerides were evaluated before and after treatment in the *C. chilense* groups. Lower doses of *C. chilense* were associated with modest numerical reductions in fecal egg counts, whereas the highest dose showed greater variability and lower apparent efficacy. However, no statistically significant overall treatment effect was detected. In an exploratory analysis, parasite type was associated with variation in post-treatment egg counts. Biochemical analyses did not show a consistent dose-dependent pattern compatible with overt hepatotoxicity. These results suggest that *C. chilense* may have preliminary potential as a complementary phytotherapeutic candidate, but larger studies with improved sampling design, standardized phytochemical characterization, and greater statistical power are needed.

## Simple Summary

Gastrointestinal parasites are a major problem in sheep production and are commonly controlled with synthetic antiparasitic drugs. However, drug resistance and environmental concerns have increased interest in plant-based alternatives. In this exploratory study, we evaluated whether *Chenopodium chilense* (“paico”), a plant species traditionally used in Chilean medicine, could reduce parasite egg counts in sheep. Thirty adult ewes received either commercial antiparasitic drugs or different oral doses of *C. chilense*. Lower doses of *C. chilense* were associated with modest numerical reductions in fecal egg counts, although the differences were not statistically significant. The highest dose showed greater variability and lower apparent efficacy. Blood biochemical analyses did not reveal a consistent pattern suggestive of dose-dependent liver toxicity. These findings suggest that *C. chilense* may deserve further study as a complementary phytotherapeutic option within integrated parasite-control strategies, but the present results do not support its use as a replacement for conventional anthelmintics.

## 1. Introduction

Gastrointestinal parasitism remains one of the main health constraints affecting sheep production systems worldwide, particularly in extensive and semi-extensive systems. These infections negatively affect animal welfare, productivity, and farm profitability and represent a persistent challenge for sustainable small-ruminant production systems [1,2,3]. In southern Chile, sheep production plays a central socio-economic role within family farming systems, where parasitic diseases continue to be a major limitation to sustainable livestock production [4].

The control of gastrointestinal parasites in sheep has traditionally relied on the systematic use of broad-spectrum anthelmintic drugs, including benzimidazoles, imidazothiazoles, salicylanilides, and macrocyclic lactones. However, the widespread and often indiscriminate use of these compounds has led to increasing levels of anthelmintic resistance in nematode populations, reducing treatment efficacy and threatening the long-term sustainability of parasite control programs [5,6,7]. Anthelmintic resistance is widely recognized as a heritable and persistent phenomenon driven by selection pressure and the spread of resistant parasite genotypes within populations [8,9].

Beyond resistance, increasing attention has been given to the environmental impact of conventional antiparasitic drugs. Macrocyclic lactones, such as ivermectin and moxidectin, are excreted largely unmetabolized and persist in dung and soil, exerting toxic effects on non-target organisms, particularly coprophagous insects and aquatic invertebrates [10,11,12,13]. These effects compromise key ecosystem services, including dung degradation, nutrient recycling, soil structure, and biological control of pest species, ultimately affecting pasture productivity and environmental sustainability [14,15,16,17]. This evidence highlights the need for alternative parasite control strategies that are both effective and environmentally responsible, in line with the One Health framework.

In this context, medicinal plants have emerged as promising sources of bioactive compounds with antiparasitic potential. The genus Chenopodium includes numerous species distributed worldwide, several of which have been traditionally used for gastrointestinal disorders and parasitic conditions in humans and animals [18]. Experimental studies have demonstrated anthelmintic activity in several species of this genus. Notably, *Chenopodium album* has shown significant reductions in fecal egg counts of trichostrongylid nematodes in sheep under controlled conditions [19], while Chenopodium ambrosioides has been traditionally recognized for vermifugal properties associated with its essential oil composition [20,21].

The anthelmintic activity of *Chenopodium* species has been primarily attributed to secondary metabolites such as ascaridole, a monoterpene peroxide with documented nematocidal and antiprotozoal activity [20,22]. However, the use of *C. ambrosioides* has been limited by reports of toxicity, which restrict its application in veterinary medicine [23]. In contrast, *Chenopodium chilense Schrad*., commonly known as “paico” in Chile, is a native species traditionally used in Mapuche medicine for gastrointestinal ailments and spasmodic disorders [24]. Previous phytochemical and biological studies have suggested that *C. chilense* may have therapeutic potential and a more favorable safety profile than some closely related species [25].

Despite its ethnobotanical relevance and favorable safety profile, *C. chilense* remains poorly studied as a natural anthelmintic in livestock. Since its botanical description [26] and the initial chemical and biological characterization by [25], no controlled in vivo studies have systematically evaluated its efficacy against gastrointestinal parasites in sheep. This lack of experimental evidence represents a critical knowledge gap that limits the scientific validation and potential incorporation of this native species into sustainable parasite control programs for small ruminant production systems.

Therefore, the aim of the present exploratory study was to assess whether *Chenopodium chilense* was associated with changes in gastrointestinal parasite burden in sheep from southern Chile and to compare the observed response with that of conventional anthelmintic treatments.

## 2. Materials and Methods

The study was conducted at the Maquehue Experimental Center of the Universidad de la Frontera, Temuco, Chile (38°44′ S, 72°35″ W). A total of 30 adult female Criollo Araucana sheep (2–4 years old; body weight range 43–68 kg) were included in the study. Animals were allocated into five experimental groups (n = 6 per group). Group allocation was performed after baseline coprological screening, with an attempt to minimize differences in initial EPG values among groups. The study protocol was reviewed and approved by the Ethics Scientific Committee, University of La Frontera, under approval number N°060/24. All procedures involving animals were conducted in accordance with institutional guidelines for animal care and use.

### 2.1. Experimental Design and Treatments

Whole plants of *Chenopodium chilense Schrad.* (stems and leaves) were collected from field-grown plants in Radal, Araucanía, Chile, during the 2025 growing season. The collected material consisted of aerial plant parts, which were dehydrated at 40 °C until constant weight was achieved and then ground into a fine powder. A single batch of dried plant material was used throughout the experimental period in order to reduce within-study variability. However, detailed information on soil characteristics, plant age, phenological stage, and possible variation among plant populations was not available. These factors were therefore considered potential sources of variability and are explicitly acknowledged in the Discussion as limitations of the study. The powdered material was reconstituted in water immediately before administration to obtain an aqueous paste, which was administered using an oro-ruminal tube to ensure accurate delivery of the assigned dose (g/kg body weight).

Prior to treatment allocation, animals were screened for gastrointestinal parasite burden using a coprological examination based on the McMaster technique. Animals with detectable gastrointestinal parasitism were selected on the basis of coprological screening. According to the available farm records and information provided by the animal owners, the animals had not received anthelmintic treatment for at least six months prior to the beginning of the study. Although an effort was made to minimize baseline differences among groups, some between-group variation in initial EPG values remained. Before treatment, all animals were clinically inspected and considered apparently healthy, with no overt signs of systemic disease. Animals were maintained under similar management and nutritional conditions throughout the study.

Following baseline assessment, animals were assigned to the following treatments:

• T1: Oral administration of a commercial anthelmintic (Rumiten^®,^,nDragpharma, Santiago, Chile) at 3.75 mg/kg praziquantel and 5 mg/kg fenbendazole.
• T2: Subcutaneous administration of a commercial anthelmintic (Invermic Plus^®^, Dragpharma, Santiago, Chile) at 0.2 mg/kg ivermectin and 5 mg/kg closantel.
• T3: Oral administration of *C. chilense* at 0.5 g dry matter/kg body weight.
• T4: Oral administration of *C. chilense* at 1.0 g dry matter/kg body weight.
• T5: Oral administration of *C. chilense* at 2.0 g dry matter/kg body weight.

After oro-ruminal administration, animals were clinically observed for overt signs of acute intolerance, including abnormal behavior, excessive salivation, regurgitation, depression, or visible discomfort.

### 2.2. Coprological Analysis

After treatment administration, fecal samples were collected over a three-week follow-up period on predefined non-consecutive days each week (Monday, Wednesday, and Friday) to account for intermittent egg shedding. Samples (~5 g) were obtained by transrectal palpation. Egg counts were determined using the McMaster technique and expressed as EPG.

### 2.3. Outcome Measures

The primary outcome was the post-treatment change in fecal egg counts (EPG) relative to baseline within and among treatment groups. Secondary outcomes included comparison of the anthelmintic efficacy of *C. chilense* relative to commercial anthelmintics and assessment of the dose–response relationship of *C. chilense* for gastrointestinal parasite control in sheep.

### 2.4. Serum Biochemical Analysis

Blood samples were collected before and after treatment administration to evaluate serum biochemical variables associated with hepatic function and metabolism. Serum activities of aspartate aminotransferase (AST), gamma-glutamyl transferase (GGT), and alkaline phosphatase (ALP), as well as serum concentrations of total protein (TP), albumin (ALB), total cholesterol (TC), and triglycerides (TG), were determined using standard enzymatic–colorimetric methods according to the manufacturer’s instructions. Analyses were performed using an automated clinical chemistry analyzer, Metrolab 1600 DR-K, Wiener lab., Buenos Aires, Argentina). The biochemical determinations were conducted under routine veterinary diagnostic laboratory procedures.

### 2.5. Statistical Analysis

To explore differences among treatment groups, a one-way analysis of variance (ANOVA) was applied to post-treatment EPG values and pre–post changes, using Statgraphics Centurion XIX. Statistical significance was set at *p* < 0.05.

In addition, a multifactorial ANOVA was used as an exploratory analysis to assess whether treatment group, parasite type, and their interaction were associated with variation in post-treatment EPG values. This model included treatment type and parasite type as fixed factors in order to evaluate potential interactions between them and to determine their combined effect on total parasitic burden.

These analyses aimed to compare the observed antiparasitic responses among *C. chilense* doses and commercial treatments and explore possible non-linear dose-related patterns.

To evaluate potential dose-dependent hepatotoxic effects in treated sheep, serum concentrations of AST, GGT, ALP, TP, albumin, total cholesterol, and triglycerides were analyzed using linear mixed-effects models for repeated measures. A separate model was fitted for each analyte, including treatment group, sampling time (before and after administration), and their interaction as fixed effects. Animal was included as a random effect to account for the non-independence of repeated observations obtained from the same individual. The significance of the group × time interaction was used to determine whether the biochemical response to treatment differed among dose levels, thereby identifying potential treatment-associated hepatic alterations. Descriptive results are presented as mean ± standard deviation, and statistical significance was declared at *p* < 0.05.

## 3. Results

### 3.1. Baseline Parasitic Load Prior to Treatment

To characterize baseline parasitic conditions, mean fecal egg counts (EPG) and standard deviations (SDs) were calculated for the main parasitic taxa detected prior to treatment administration. These analyses provided a descriptive overview of pre-treatment parasitic load before experimental intervention.

Table 1, Table 2 and Table 3 summarize the mean (±SD) EPG values for *Strongylo* spp., *Coccidias* spp., and *Nematodirus* spp. at the two pre-treatment sampling times. T0 corresponded to the initial screening performed during animal selection, while T0.2 corresponded to the sampling conducted immediately before treatment administration.

Across sampling times, parasitic loads remained low and stable for all parasite groups. *Strongylo* spp. showed mean values of 4.0 ± 5.81 EPG at T0 and 4.8 ± 7.09 EPG at T0.2, while *Coccidias* spp. and *Nematodirus* spp. presented consistently low mean counts. These results suggested relatively low and stable pre-treatment counts for the parasite taxa evaluated at the screening stage; however, variation among treatment groups remained evident in the broader pre-treatment dataset.

### 3.2. Comparison of Parasitic Load Before and After Treatment

Mean EPG values before (T0) and after treatment were calculated for each experimental group to describe general trends in parasitic load reduction.

A numerical reduction in mean parasitic load was observed across all treatments. This numerical decrease appeared greater in animals receiving lower doses of *C. chilense* (0.5–1.0 g/kg) and in those treated with commercial anthelmintics. However, large standard deviations indicated high individual variability, particularly in the 2.0 g/kg *C. chilense* group.

### 3.3. One-Way ANOVA Between Pre- and Post-Treatment Parasitic Load

A one-way ANOVA was performed to explore differences in EPG values between the pre-treatment and post-treatment measurements.

No statistically significant differences were detected between initial and post-treatment parasitic loads (*p* > 0.05). The lack of significance was associated with high within-group variability, particularly in treatments involving higher doses of *C. chilense * (Table 4).

**Table 4:** Multifactorial ANOVA results for fecal egg counts (EPG).

Factor	F-Value	*p*-Value	Significance
Treatment	1.44	0.2294	Not significant
Parasite type	3.57	0.0325	Significant
Treatment × Parasite	1.0	>0.05	Not significant

Post hoc analysis using Fisher’s LSD test grouped treatments into two homogeneous categories. Lower doses of *C. chilense* (0.5–1.0 g/kg) showed descriptively lower post-treatment EPG values than the highest dose group and overlapped with the range observed in animals treated with commercial anthelmintics; however, these differences should be interpreted cautiously, given the lack of a significant overall treatment effect.

### 3.4. Multifactorial ANOVA: Effects of Treatment and Parasite Type

Given the absence of significant differences in the one-way ANOVA and the observed variability among parasite taxa, a multifactorial ANOVA was conducted. Parasitic load (EPG) was considered the dependent variable, while treatment type and parasite species were included as fixed factors.

Parasite type had a significant effect on post-treatment parasitic load (*p* = 0.0325), whereas treatment type and the interaction between treatment and parasite were not statistically significant. These exploratory results suggest that variation in post-treatment parasitic burden was more strongly associated with parasite type than with treatment group.

### 3.5. Duncan’s Multiple Range Test by Parasite Type

Duncan’s multiple range test was applied to identify differences among parasite taxa (Table 5).

**Table 5:** Mean fecal egg counts (EPG) and homogeneous groups according to Duncan’s multiple range test.

Parasite	Mean EPG	Homogeneous Group
*Nematodirus* spp.	0.67	A
*Coccidias* spp.	1.97	A
*Strongylo* spp.	5.67	B

Parasites sharing the same letter did not differ significantly (*p* > 0.05). *Strongylo* spp. formed a distinct group with significantly higher EPG values compared to *Nematodirus* spp. and *Coccidias* spp., which did not differ from each other.

Baseline parasitic loads appeared broadly comparable at screening, although variation in initial EPG values among treatment groups remained evident in the pre-treatment dataset. Although one-way ANOVA did not detect statistically significant differences among treatments, a consistent trend toward parasitic load reduction was observed in animals treated with lower doses of *C. chilense* (0.5–1.0 g/kg), with post-treatment values numerically overlapping those observed in animals treated with commercial anthelmintics. Multifactorial analysis demonstrated that parasite species was the primary determinant of post-treatment parasitic load, with *Strongylo* spp. showing greater persistence relative to other taxa.

Finally, based on the reference intervals and the pre- versus post-treatment comparisons, no clear biochemical evidence of dose-dependent hepatotoxicity was observed in the treated sheep. Although some analytes, particularly GGT and triglycerides, were above reference values, these alterations were already present at baseline and did not show a consistent post-treatment worsening pattern. Moreover, the only significant group × time interaction was detected for ALP, but its direction was not compatible with a clear dose-dependent toxic effect.

## 4. Discussion

The present study aimed to evaluate the efficacy of *Chenopodium chilense* as a natural alternative for the control of gastrointestinal parasites in sheep from southern Chile. Overall, the results suggest that oral administration of *C. chilense*, particularly at 0.5 and 1.0 g/kg body weight, may be associated with a modest reduction in fecal egg counts. However, these reductions did not reach statistical significance (*p* > 0.05) when compared with conventional anthelmintic treatments, which exhibited a more pronounced and consistent decrease in parasitic load.

Although these reductions did not reach statistical significance, the descriptive pattern observed in the lower-dose groups may be compatible with a modest biological effect that warrants confirmation in larger studies. Descriptively, lower post-treatment counts were observed for *Coccidias* spp. and *Nematodirus* spp. than for *Strongylo* spp.; however, this pattern should be interpreted cautiously because treatment effects were not statistically significant. These findings were consistent with the multifactorial ANOVA results, which identified parasite type as the primary determinant of post-treatment parasitic burden, rather than treatment type or treatment–parasite interaction.

Although the one-way ANOVA did not detect statistically significant differences among treatments (*p* > 0.05), consistent trends toward reduced fecal egg counts were observed, particularly at lower doses of *C. chilense*. Given the inherent biological variability of fecal egg count data and the limited sample size, the absence of statistical significance does not necessarily exclude the possibility of a modest biological effect, particularly in exploratory studies with limited sample size and substantial inter-individual variability [27,28]. In this context, the multifactorial ANOVA provided additional insight by identifying parasite type as a significant determinant of post-treatment parasitic burden, highlighting the importance of species-specific responses in the interpretation of treatment efficacy.

Therefore, statistical outcomes were interpreted in conjunction with descriptive trends and biological plausibility, rather than as sole indicators of treatment effectiveness. This approach is consistent with previous studies evaluating plant-derived anthelmintic compounds under experimental conditions with high inter-individual variability, and differences in phytochemical composition may affect treatment response [19,29,30].

The moderate and selective antiparasitic activity observed for *C. chilense* was in agreement with previous studies conducted on species of the genus Chenopodium. Previous studies on *Chenopodium* spp. suggest that part of their antiparasitic activity may be related to bioactive compounds such as ascaridole and other secondary metabolites capable of interfering with parasite physiology [20,22]. However, the active ingredient of the *C. chilense* material evaluated in this study was not identified. Based on previous evidence from related *Chenopodium* species, ascaridole and other monoterpenes may represent candidate bioactive compounds; however, this was not confirmed in the plant material used in the present trial. Therefore, the mechanistic interpretation of the observed reductions in fecal egg counts should be considered indirect and hypothesis-generating. The high inter-individual variability detected, particularly at the highest dose (2.0 g/kg), likely limited the detection of statistically significant differences.

Comparison among *C. chilense* doses suggested a possible non-linear response pattern. Lower doses (0.5–1.0 g/kg) showed post-treatment values that numerically overlapped those observed in the commercial-treatment groups, whereas the highest dose (2.0 g/kg) exhibited greater dispersion and reduced apparent efficacy. This pattern may be compatible with a non-linear dose–response relationship; however, the limited sample size and high variability prevent firm conclusions regarding a true biphasic response [29]. In complex plant-derived preparations, reduced efficacy at higher doses may be associated with hormetic responses, dose-dependent limitations in intestinal absorption and metabolism, and antagonistic interactions among bioactive constituents within the extract [30,31]. No overt behavioral abnormalities or clinical signs of acute intolerance were observed during or after oro-ruminal administration, including in animals receiving 2.0 g/kg. However, palatability was not directly evaluated because the preparation was administered via oro-ruminal tube rather than offered voluntarily. Therefore, possible effects of handling stress, administration volume, paste consistency, ruminal distribution, or individual variation in tolerance cannot be excluded. The lower apparent efficacy and greater variability observed at 2.0 g/kg should therefore be interpreted cautiously and not attributed exclusively to a hormetic response. The lower apparent response observed at the highest dose may also reflect host-related physiological factors affecting phytochemical bioavailability. In ruminants, ruminal fermentation and high microbial activity can degrade phytochemicals before intestinal absorption, thereby reducing bioavailability. Additionally, rapid hepatic biotransformation and clearance of secondary metabolites may further limit systemic exposure to active compounds. These mechanisms have been reported for essential oils and phenolic compounds and may have contributed to the irregular response observed at the highest *C. chilense* dose [30,31].

Another relevant consideration is that the post-treatment sampling schedule may not have been optimally aligned with standard FECRT time points for detecting maximal reductions in egg shedding. Previous studies evaluating *Chenopodium* spp. have used follow-up schedules that capture delayed or cumulative responses, and current FECRT guidelines recommend post-treatment assessment at specific time points depending on the anthelmintic class evaluated [27,28]. Therefore, the sampling design used here may have limited detection of the full temporal response. The three-week follow-up period with non-consecutive sampling allowed exploratory monitoring of post-treatment egg shedding but may not have fully captured the temporal dynamics of a plant-based intervention. Unlike synthetic anthelmintics, which often produce more rapid and direct effects, botanical preparations may act more slowly or require repeated administration to generate measurable changes. Therefore, future studies should include longer monitoring periods, standardized post-treatment FECRT time points, and repeated-dose strategies to better characterize the temporal response to *C. chilense*.

In contrast, the larger and more consistent numerical reductions observed in animals treated with commercial anthelmintics were expected and are consistent with the well-characterized pharmacological mechanisms of these drugs. Several conventional anthelmintic classes act directly on parasite neuromuscular function; for example, levamisole, pyrantel, and morantel act as agonists of nicotinic acetylcholine receptors in nematode muscle, inducing spastic paralysis, whereas macrocyclic lactones interfere with inhibitory neurotransmission through glutamate-gated chloride channels, leading to paralysis and death of the parasite [32,33]. In addition, benzimidazoles disrupt microtubule formation by binding parasite β-tubulin, which impairs essential cellular processes, including glucose uptake and energy metabolism [34,35]. Together, these specific and potent mechanisms help explain the lower variability and stronger efficacy observed for commercial anthelmintics relative to plant-based treatments.

Another relevant factor that may have influenced treatment response is the previous anthelmintic exposure of the animals. Detailed individual records of prior anthelmintic treatments were not available, and no formal previous assessment of anthelmintic resistance had been conducted in the flock. Therefore, possible previous exposure to the same or related drug classes, as well as undetected resistance to the commercial anthelmintics used in this study, cannot be excluded. This limitation is important because reduced efficacy of conventional anthelmintics may be associated not only with treatment failure but also with pre-existing resistance, reinfection dynamics, parasite species composition, or suboptimal timing of post-treatment evaluation [5,7,27,28].

Taken together, the results suggest that *C. chilense* may exert a modest antiparasitic effect, particularly at lower doses, although this interpretation should be considered preliminary. Although its efficacy was inferior to that of conventional anthelmintics, the observed trends may be compatible with selective differences among parasite taxa and a possible dose-related response, comparable to that reported for other *Chenopodium* species. These findings support further investigation of *C. chilense* as a complementary phytotherapeutic candidate within integrated parasite management programs, rather than as a replacement for conventional treatments.

The aqueous paste administered via oro-ruminal tube was used only as an experimental method to ensure accurate individual dosing and should not be interpreted as a directly recommended farm-level strategy. For practical implementation, future development would require more feasible formulations, such as standardized dried plant material, oral drenches, capsules, or pelletized supplements, together with dose standardization and safety validation [29,30]. Therefore, *C. chilense* should currently be considered only a preliminary phytotherapeutic candidate for further research within integrated parasite-control programs, rather than an immediately applicable replacement for conventional anthelmintics.

Future studies should consider longer post-treatment monitoring periods, larger sample sizes, repeated dosing strategies, and characterization of phytochemical profiles to better elucidate the antiparasitic potential and practical applicability of *C. chilense* in small ruminant production systems.

Overall, the biochemical findings did not support a consistent dose-dependent hepatotoxic effect of *C. chilense* under the conditions evaluated (Table 6). Among the evaluated variables, only ALP showed a significant group × time interaction, whereas AST, GGT, total protein, albumin, total cholesterol, and triglycerides did not exhibit significant dose-dependent pre–post changes. In veterinary clinical pathology, the interpretation of possible hepatobiliary injury should be based on the combined behavior of several biochemical analytes rather than on an isolated alteration in a single marker, because increases in hepatic enzymes indicate hepatic abnormality but do not by themselves define the severity of liver dysfunction [36]. Before treatment, all animals were clinically inspected and considered apparently healthy, with no overt signs of systemic disease. They were maintained under similar management and nutritional conditions. However, some biochemical analytes, particularly GGT and triglycerides, were above reference values at baseline, indicating that pre-existing metabolic or hepatobiliary variation cannot be excluded. These baseline alterations may have influenced individual biochemical responses and represent a limitation of the safety assessment. Nevertheless, because these deviations were present before treatment and did not show a consistent dose-dependent post-treatment worsening, the results do not support a clear pattern of treatment-associated hepatotoxicity. Moreover, AST is not liver-specific because it is also present in skeletal muscle and other tissues, whereas GGT and ALP are more commonly associated with cholestatic or biliary disease; importantly, ALP may also originate from non-hepatic tissues such as bone and intestine, which limits the interpretation of an isolated increase [36,37]. In addition, evidence of clinically relevant hepatic dysfunction is usually accompanied by coordinated alterations in variables related to hepatic synthetic or metabolic capacity, including albumin and other biochemical indicators, rather than by a single inconsistent enzymatic fluctuation [36,37]. Therefore, the ALP response observed here was not biologically consistent with a toxic dose–response pattern, since the intermediate-dose group exhibited increased levels, whereas it decreased in the highest-dose group after treatment. Taken together, these findings indicate that the tested doses were not associated with a consistent biochemical pattern suggestive of overt hepatotoxicity based on routine serum analytes [36].

**Table 6:** Effects of three treatment doses on serum biochemical variables associated with hepatic function and metabolism in sheep before and after administration.

	Group 1 (0.5 g/kg) Pre			Group 2 (1 g/kg) Pre			Group 3 (2 g/kg) Pre			
Variable	Pre	Post	Δ (Post−Pre)	Pre	Post	Δ (Post−Pre)	Pre	Post	Δ (Post−Pre)	Group × Time *p*-Value
AST (U/L)	89.56 ± 39.40	69.52 ± 32.31	−20.04 ± 27.63	89.37 ± 28.17	85.57 ± 48.33	−3.80 ± 43.25	92.32 ± 28.55	89.30 ± 45.27	−3.02 ± 50.96	0.548
GGT (U/L)	44.58 ± 11.48	56.34 ± 11.34	11.76 ± 15.28	56.37 ± 32.08	53.83 ± 19.36	−2.53 ± 30.29	45.18 ± 15.48	50.08 ± 14.47	4.90 ± 21.78	0.528
ALP (U/L)	216.27 ± 94.05	207.80 ± 179.30	−8.47 ± 118.93	244.99 ± 49.54	347.76 ± 138.11	102.77 ± 136.21	385.32 ± 162.91	200.24 ± 54.87	−185.08 ± 168.09	0.013
TP (g/L)	80.50 ± 8.06	79.90 ± 16.18	−0.60 ± 16.27	83.50 ± 11.37	80.85 ± 5.67	−2.65 ± 10.77	84.38 ± 7.65	88.53 ± 18.17	4.15 ± 19.24	0.547
ALB (g/L)	31.38 ± 3.80	37.36 ± 2.40	5.98 ± 4.19	35.93 ± 5.96	35.82 ± 1.75	−0.12 ± 5.12	38.02 ± 5.03	39.23 ± 5.96	1.22 ± 7.20	0.330
TC (mmol/L)	2.72 ± 1.02	3.12 ± 2.18	0.40 ± 1.79	2.43 ± 1.17	2.35 ± 0.68	−0.08 ± 1.33	2.18 ± 0.40	3.63 ± 1.64	1.45 ± 1.56	0.213
TG (mmol/L)	1.82 ± 0.40	1.20 ± 0.50	−0.62 ± 0.43	1.50 ± 0.84	1.77 ± 0.92	0.27 ± 1.29	1.62 ± 0.60	1.63 ± 1.01	0.02 ± 1.09	0.656

Data are presented as mean ± SD. Groups received dried *Chenopodium chilense* at 0.5, 1.0, or 2.0 g/kg of live weight. Pre and Post indicate values before and after treatment administration, respectively. Δ was calculated as Post−Pre. AST: aspartate aminotransferase; GGT: gamma-glutamyl transferase; ALP: alkaline phosphatase; TP: total protein; ALB: albumin; TC: total cholesterol; TG: triglycerides. The group × time *p*-value was obtained from a repeated-measures mixed model. Significance was set at *p* < 0.05. Data are presented as mean ± standard deviation. Group 1: 0.5 g/kg; Group 2: 1 g/kg; Group 3: 2 g/kg. Δ represents the absolute difference between post-treatment and pre-treatment values (Post−Pre). The effect of treatment was evaluated using a linear mixed-effects model for repeated measures, including treatment group, sampling time, and the group × time interaction as fixed effects, and animal as a random effect. The group × time interaction was used to determine whether the biochemical response differed according to dose, as an indicator of potential dose-dependent hepatotoxicity in treated sheep.

From a clinical pathology perspective, liver toxicity is usually supported by a coordinated increase in markers of hepatocellular leakage or cholestasis, often accompanied by changes in variables associated with hepatic synthetic function [36,37]. That pattern was not observed here. AST remained relatively stable across groups, while total protein and albumin were maintained within their reference intervals, arguing against a marked impairment of hepatic function. Likewise, although GGT exceeded the reference limit in several animals, this alteration was already present before treatment and did not show a consistent post-treatment worsening. Therefore, the available biochemical evidence does not suggest detectable liver injury based on the routine serum analytes evaluated in this study.

These results are relevant in the current context of sheep production, where there is increasing interest in plant-derived antiparasitic alternatives. Anthelmintic resistance in ovine nematodes has been recognized in southern Latin America for decades and remains an important challenge for parasite control programs [5,6,7,9]. This problem is especially important in sheep systems, given the continuing productive relevance of small ruminants in animal agriculture [3] . In Chile, sheep production also intersects with endemic parasitic problems of veterinary and public health importance, including cystic echinococcosis [1,2]. Within this scenario, botanicals such as paico are attractive because they may contribute to more diversified and locally adapted parasite-control strategies.

The biological plausibility of paico as a phytotherapeutic candidate is supported by the known medicinal relevance of the genus *Chenopodium*. In Chile, *Chenopodium chilense* has been identified as paico, and its biological and chemical properties have been described previously [25]. More broadly, Chenopodium species are recognized as important medicinal and ethnobotanical resources, including in traditional practices in Araucanía, Chile [18,20,21,24,26]. In addition, evidence from sheep indicates that Chenopodium album has in vitro and in vivo anthelmintic activity against trichostrongylid nematodes, supporting the broader antiparasitic potential of this botanical group [19].

At the same time, safety assessment is essential because Chenopodium-derived preparations may contain bioactive compounds with toxic potential. In particular, ascaridole has been widely associated with the pharmacological activity of *Chenopodium* spp. and is also considered a plausible contributor to toxicity, especially at unsuitable doses or in concentrated preparations [20,21,22]. For that reason, the absence of a clear biochemical signature of liver damage in the present study is encouraging. However, this should be interpreted as preliminary evidence of tolerability rather than definitive proof of safety because routine serum biochemistry may fail to detect subtle, early, or focal lesions.

The interest in paico-based alternatives is also strengthened by the limitations of conventional endectocides. Macrocyclic lactones remain highly useful antiparasitic drugs, but their environmental externalities are well documented. Ivermectin and related compounds can persist in excreta and negatively affect non-target organisms associated with dung, soil, sediment, and aquatic environments [11,12,13,16]. Chronic toxicity of ivermectin has been demonstrated in benthic invertebrates such as Chironomus riparius and Lumbriculus variegatus [14], and macrocyclic lactones such as moxidectin can also accumulate in water–sediment systems and affect aquatic and benthic communities [15]. In terrestrial systems, endectocide residues have been shown to reduce the survival of dung-associated beetles, as summarized in a meta-analysis by [10], and antiparasitic management can alter dung beetle assemblages and their functional structure [17]. Thus, the search for effective and safe plant-based antiparasitic strategies is justified not only by resistance concerns but also by the need to reduce undesirable ecological effects associated with synthetic drugs.

In summary, the present results indicate that the tested paico-based treatment did not produce clear biochemical evidence of dose-dependent hepatotoxicity in sheep based on the routine serum biochemical analytes evaluated. This is a relevant preliminary finding in the broader context of developing alternative antiparasitic approaches for small-ruminant production systems affected by resistance and sustainability constraints. Future studies should combine efficacy testing against gastrointestinal nematodes with standardized phytochemical characterization and broader toxicological evaluation in order to determine whether paico can be incorporated safely into integrated parasite-control programs [5,11,19].

### Study Limitations

First, the present study was conducted as an exploratory in vivo trial, and the sample size (n = 6 animals per treatment) represents a methodological limitation that may have reduced the statistical power to detect significant differences among treatments. While this number of animals is consistent with preliminary studies evaluating phytotherapeutic alternatives under controlled experimental conditions, the relatively small group size likely contributed to the high within-group variability observed in fecal egg counts, particularly at higher doses of *Chenopodium chilense*. Consequently, the results should be interpreted as indicative of biological trends rather than definitive evidence of efficacy, and conclusions regarding treatment effectiveness should be considered preliminary.

Second, baseline EPG values differed among treatment groups despite attempts to minimize initial differences during allocation. This imbalance may have influenced the apparent magnitude of post-treatment reductions, and it limits direct comparison among treatments. ANCOVA would be appropriate in a larger confirmatory trial, but in the present exploratory study, the small sample size and high baseline dispersion would have produced unstable adjusted estimates. Therefore, treatment comparisons should be interpreted as exploratory and descriptive rather than as definitive evidence of efficacy.

Third, the administered batch of *C. chilense* was not chemically characterized by HPLC, GC-MS, or related analytical methods. Therefore, the presence and concentration of ascaridole or other potentially active secondary metabolites were not confirmed in the plant material used in this trial. The mechanistic interpretation based on Chenopodium-derived compounds should therefore be considered indirect and based on previous literature for the genus. This lack of phytochemical standardization limits reproducibility and may have contributed to the observed variability in biological response.

Fourth, the toxicological assessment relied on routine serum biochemical markers only; a more robust evaluation would require integration with additional indicators such as bilirubin, GLDH, SDH, oxidative stress markers, clinical findings, and histopathology.

Fifth, some analytes, particularly GGT and triglycerides, were already above reference limits at baseline, making it difficult to attribute isolated deviations exclusively to treatment. Under these conditions, the lack of a coherent post-treatment deterioration across dose groups becomes more informative than isolated statistical differences.

Sixth, the lack of standardized phytochemical characterization of the administered plant material limits the interpretation of dose-related effects and reduces reproducibility across studies.

## 5. Conclusions

Oral administration of *Chenopodium chilense* was associated with a modest numerical reduction in fecal egg counts in treated sheep, particularly at the lower dose levels. However, the observed differences were not statistically significant, and the marked inter-individual variability limits definitive conclusions regarding efficacy. Parasite type appeared to influence the post-treatment parasitic profile, with *Strongylo* spp. showing greater persistence than the other taxa evaluated. Under the conditions of this exploratory study, routine serum biochemical analytes did not indicate a consistent dose-dependent hepatotoxic effect. Overall, these findings support further investigation of *C. chilense* as a complementary phytotherapeutic candidate within integrated parasite-control strategies, but not as a replacement for conventional anthelmintics at this stage.

## Figures and Tables

**Table 1:** Mean fecal egg counts (EPG; mean ± SD) for *Strongyloides* spp., coccidia, and *Nematodirus* spp. during pre-treatment sampling.

Parasite	T0	T0.2
*Strongyloides* spp.	4.00 ± 5.81	4.80 ± 7.09
Coccidia	3.53 ± 5.11	2.67 ± 2.82
*Nematodirus* spp.	0.33 ± 0.33	1.29 ± 1.29

EPG: eggs per gram of feces; SD: standard deviation. T0: initial coprological screening performed during animal selection; T0.2: second pre-treatment sampling performed immediately before treatment administration. Values are presented as mean ± SD. No treatment was administered at either sampling point.

**Table 2:** Mean fecal egg counts (EPG; mean ± SD) before and after treatment according to treatment group.

Treatment	Initial EPG	Post-Treatment EPG	Absolute Reduction	Reduction (%)
*C. chilense* 0.5 g/kg of LW	950.0 ± 620.3	733.3 ± 703.3	216.7	22.8
*C. chilense* 1.0 g/kg of LW	916.7 ± 704.5	650.0 ± 674.5	266.7	29.1
*C. chilense* 2.0 g/kg of LW	2050.0 ± 3100.8	1916.7 ± 3210.9	133.3	6.5
Invermic Plus^®^	816.7 ± 310.2	616.7 ± 318.8	200.0	24.5
Rumiten^®^	400.0 ± 110.1	233.3 ± 103.3	166.7	41.7

EPG: eggs per gram of feces; LW: live weight; SD: standard deviation. Initial EPG: fecal egg counts measured before treatment administration; post-treatment EPG: fecal egg counts measured after treatment. Absolute reduction was calculated as the initial EPG minus the post-treatment EPG. Reduction (%) was calculated as [(initial EPG − post-treatment EPG)/initial EPG] × 100. Invermic Plus^®^ contains ivermectin and praziquantel as active ingredients; Rumiten^®^ contains fenbendazole as an active ingredient.

**Table 3:** One-way ANOVA comparing initial and post-treatment EPG values.

Comparison	F-Value	*p*-Value	Significance
Initial vs. Post-treatment EPG	1.07	0.3916	Not significant

EPG: eggs per gram of feces. Initial EPG: fecal egg counts measured before treatment administration. Post-treatment EPG: fecal egg counts measured during the follow-up period after treatment. Statistical significance was set at *p* < 0.05.

## Data Availability

The data presented in this study are available on request from the corresponding author due to ongoing collaborative analyses with another institution.
